# Burden of cardiac arrhythmias in patients with acute myocardial infarction and their impact on hospitalization outcomes: insights from China acute myocardial infarction (CAMI) registry

**DOI:** 10.1186/s12872-024-03889-w

**Published:** 2024-04-23

**Authors:** Xu Xu, Zhao Wang, Jingang Yang, Xiaohan Fan, Yuejin Yang

**Affiliations:** 1 grid.506261.6 0000 0001 0706 7839 Department of Cardiology, Cardiac Arrhythmia Center, Fuwai Hospital, National Center for Cardiovascular Diseases, State Key Laboratory of Cardiovascular Disease, Chinese Academy of Medical Sciences, Peking Union Medical College, Beijing, China; 2 grid.506261.6 0000 0001 0706 7839 , Department of Cardiology, Coronary Heart Disease Center, Fuwai Hospital, National Center for Cardiovascular Diseases, State Key Laboratory of Cardiovascular Disease, Chinese Academy of Medical Sciences and Peking Union Medical College, Beijing, China

**Keywords:** Acute myocardial infarction, Cardiac arrhythmias, Risk factors, Epidemiology

## Abstract

**Background:**

The coexistence of cardiac arrhythmias in patients with acute myocardial infarction (AMI) usually exhibits poor prognosis. However, there are few contemporary data available on the burden of cardiac arrhythmias in AMI patients and their impact on in-hospital outcomes.

**Methods:**

The present study analyzed data from the China Acute Myocardial Infarction (CAMI) registry involving 23,825 consecutive AMI patients admitted to 108 hospitals from January 2013 to February 2018. Cardiac arrhythmias were defined as the presence of bradyarrhythmias, sustained atrial tachyarrhythmias, and sustained ventricular tachyarrhythmias that occurred during hospitalization. In-hospital outcome was defined as a composite of all-cause mortality, cardiogenic shock, re-infarction, stroke, or heart failure.

**Results:**

Cardiac arrhythmia was presented in 1991 (8.35%) AMI patients, including 3.4% ventricular tachyarrhythmias, 2.44% bradyarrhythmias, 1.78% atrial tachyarrhythmias, and 0.73% ≥2 kinds of arrhythmias. Patients with arrhythmias were more common with ST-segment elevation myocardial infarction (83.3% vs. 75.5%, *P* < 0.001), fibrinolysis (12.8% vs. 8.0%, *P* < 0.001), and previous heart failure (3.7% vs. 1.5%, *P* < 0.001). The incidences of in-hospital outcomes were 77.0%, 50.7%, 43.5%, and 41.4%, respectively, in patients with ≥ 2 kinds of arrhythmias, ventricular tachyarrhythmias, bradyarrhythmias, and atrial tachyarrhythmias, and were significantly higher in all patients with arrhythmias than those without arrhythmias (48.9% vs. 12.5%, *P* < 0.001). The presence of any kinds of arrhythmia was independently associated with an increased risk of hospitalization outcome (≥ 2 kinds of arrhythmias, OR 26.83, 95%CI 18.51–38.90; ventricular tachyarrhythmias, OR 8.56, 95%CI 7.34–9.98; bradyarrhythmias, OR 5.82, 95%CI 4.87–6.95; atrial tachyarrhythmias, OR4.15, 95%CI 3.38–5.10), and in-hospital mortality (≥ 2 kinds of arrhythmias, OR 24.44, 95%CI 17.03–35.07; ventricular tachyarrhythmias, OR 13.61, 95%CI 10.87–17.05; bradyarrhythmias, OR 7.85, 95%CI 6.0-10.26; atrial tachyarrhythmias, OR 4.28, 95%CI 2.98–6.16).

**Conclusion:**

Cardiac arrhythmia commonly occurred in patients with AMI might be ventricular tachyarrhythmias, followed by bradyarrhythmias, atrial tachyarrhythmias, and ≥ 2 kinds of arrhythmias. The presence of any arrhythmias could impact poor hospitalization outcomes.

**Registration:**

Clinical Trial Registration: Identifier: NCT01874691.

## Introduction

Cardiac arrhythmias frequently complicate the clinical management of patients with acute myocardial infarction (AMI). Multiple studies have established a significant association between diverse arrhythmias with a higher morbidity and mortality in patients with AMI [1–4]. Despite the rapid advances in revascularization therapies over the last decades, managing complex arrhythmias remains challenging, particularly during the acute phase of myocardial infarction [5].

Cardiac arrhythmias complicating AMI can be classified into atrial tachyarrhythmias, ventricular tachyarrhythmias, and bradyarrhythmias. Atrial tachyarrhythmias, including atrial fibrillation and/or flutter (AF), are the most common supraventricular tachyarrhythmia complicating AMI patients [6]. It is assumed that 6-21% of AMI patients also have AF [7], whereas new-onset AF may occur in 4.5% of patients with ST-elevation myocardial infarction (STEMI) [8]. The incidence of ventricular tachyarrhythmias, such as ventricular fibrillation and ventricular flutter, has declined from 19% [9] to 3.5% [10] over thirty years in AMI patients during hospitalization. Bradyarrhythmias, including sick sinus syndrome and atrioventricular block (AVB), are relatively frequent in AMI-associated arrhythmias, particularly with inferior/posterior locations. The reported incidence of AVB in patients with AMI varies from 2.1% [11] to 6.9% [12], depending on the population and duration of observation. However, some patients with AMI may present two or more kinds of cardiac arrhythmias. With the rapid advancement of coronary intervention techniques, the incidence of cardiac arrhythmias in contemporary patients with AMI may be different from that in the past.

The impact of one specific class of cardiac arrhythmia on patients with AMI has been reported by previous studies [2, 8, 11] However, whether the presence of two or arrhythmias may demonstrate an increased risk of morbidity and mortality in patients with AMI remains unclear. Using nationally representative AMI cohorts in China, our study aimed to demonstrate the incidence of all three kinds of cardiac arrhythmias, including bradyarrhythmias, sustained atrial tachyarrhythmias, sustained ventricular tachyarrhythmias, and any two or more kinds of these cardiac arrhythmias. The impact of these cardiac arrhythmias on the in-hospital outcomes was also analyzed.

## Methods

### Study design and study population

The design of the CAMI Registry has been fully described in the previous studies [13]. Briefly, it is a prospective, nationwide, multicenter observational study involving three levels of hospitals (provincial, prefectural, and country level) in mainland China. One hundred-eight hospitals from 31 provinces and municipalities have participated in the registry. In total, 31,018 patients with a primary diagnosis of AMI who were admitted within seven days of symptoms onset were enrolled consecutively in the CAMI Registry from January 2013 to February 2018, including STEMI and non-ST-segment elevation myocardial infarction (NSTEMI). Patients’ diagnoses were made, confirmed, or revised according to the 3rd Universal Definition for Myocardial Infarction [14]. Patients were excluded if they were younger than 18 years old or older than 80 years old, had an ambiguous diagnosis, or if their medical records were unavailable.

### Definition of cardiac arrhythmias and in-hospital outcomes

The diagnosis of cardiac arrhythmias is typically recorded through electrocardiography (ECG). In cases of AMI, performing an initial ECG examination upon admission to the hospital is customary. Additionally, if fibrinolysis or emergency intervention is administered, ECG examinations are conducted within 2 h before and after the treatment. Routine ECG examinations are conducted to assess the occurrence of cardiac arrhythmia events if the patient presents with relevant symptoms. The specific class of cardiac arrhythmias in this study were defined as follows: atrial tachyarrhythmias were defined as sustained atrial flutter or atrial fibrillation, diagnosed based on criteria such as the absence of sinus P waves, irregularly irregular ventricular response, and a duration exceeding a specified time threshold. Ventricular tachyarrhythmias were sustained ventricular flutter or ventricular fibrillation, including a heart rate greater than 100 beats per minute (bpm), a QRS complex duration greater than 120 milliseconds, and a duration of at least 30 s. Bradyarrhythmias referred to severe bradycardia including second-degree type 2 atrioventricular block, third-degree atrioventricular block, and sick sinus syndrome with heart rates less than or equal to 50 beats per minutes. The presence of two or more of the above types of arrhythmias was classified as ≥ 2 kinds of arrhythmias. All the above definition of cardiac arrhythmias was confirmed to be occurred during hospitalization. If any cardiac arrhythmias were confirmed to be presented before the onset of AMI, it would be recorded as the history of one specific class of cardiac arrhythmia. All cardiac arrhythmia events are diagnosed by two experienced senior cardiologists, adhering to established guidelines [15–17].

The in-hospital outcome was a composite of all-cause mortality (defined as cardiac or non-cardiac death during hospitalization), cardiogenic shock (defined as low blood pressure, evidence of inadequate organ perfusion, and other clinical signs due to poor cardiac output), re-infarction (defined as an acute MI that occurred after initial MI with evidence of recurred ischemic symptoms, ECG changes and elevated cardiac troponin), new-onset stroke (defined as ischemic or hemorrhage stroke confirmed by CT imaging), or heart failure (diagnosed by clinical manifestations including cardiac dyspnea, pink frothy sputum, and crackles, as well as a supporting examination such as an echocardiogram, X-ray or N-terminal pro-brain natriuretic peptide) that occurred during hospitalization. Reperfusion treatment includes fibrinolysis, emergency PCI, and emergency CABG. All events were carefully checked and validated by two independent clinical physicians.

### Data collection

Data collection in this study includes patient demographics, risk factors, medical history, treatments, medications, procedures, and events. This was achieved through a secure, web-based electronic data capture system, utilizing standardized variables, predefined definitions, systematic data entry and transmission procedures, and stringent data quality control measures. The electronic database is equipped with an audit module, which performs real-time automatic checks on the completeness and basic range of data entered for all registered cases. Trained physicians at each site conducted real-time enrollment, data collection, and follow to ensure accuracy and reliability. Senior cardiologists oversaw data quality control, and hospital sites underwent random on-site audits for diagnosis and variable accuracy based on medical records. The information is collected using the standardized set of variables and standard definitions for the CAMI Registry.

### Statistical analyses

Continuous variables are expressed as median (interquartile range) and compared with the Kruskal Wallis H test. Categorical variables were expressed as percentages and compared using the chi-square test or Fisher’s exact test. Logistic regression analysis was performed to assess the association between arrhythmias and outcomes. Variables with a P-value < 0.1 in the univariate analysis and clinically significant variables were included in the multivariable analysis. All comparisons were two-sided, with statistical significance defined as *P* < 0.05. Statistical analysis was performed with SAS (version 9.4) and R (4.1.3).

## Results

### Incidence of cardiac arrhythmias in AMI patients

**Fig. 1:**
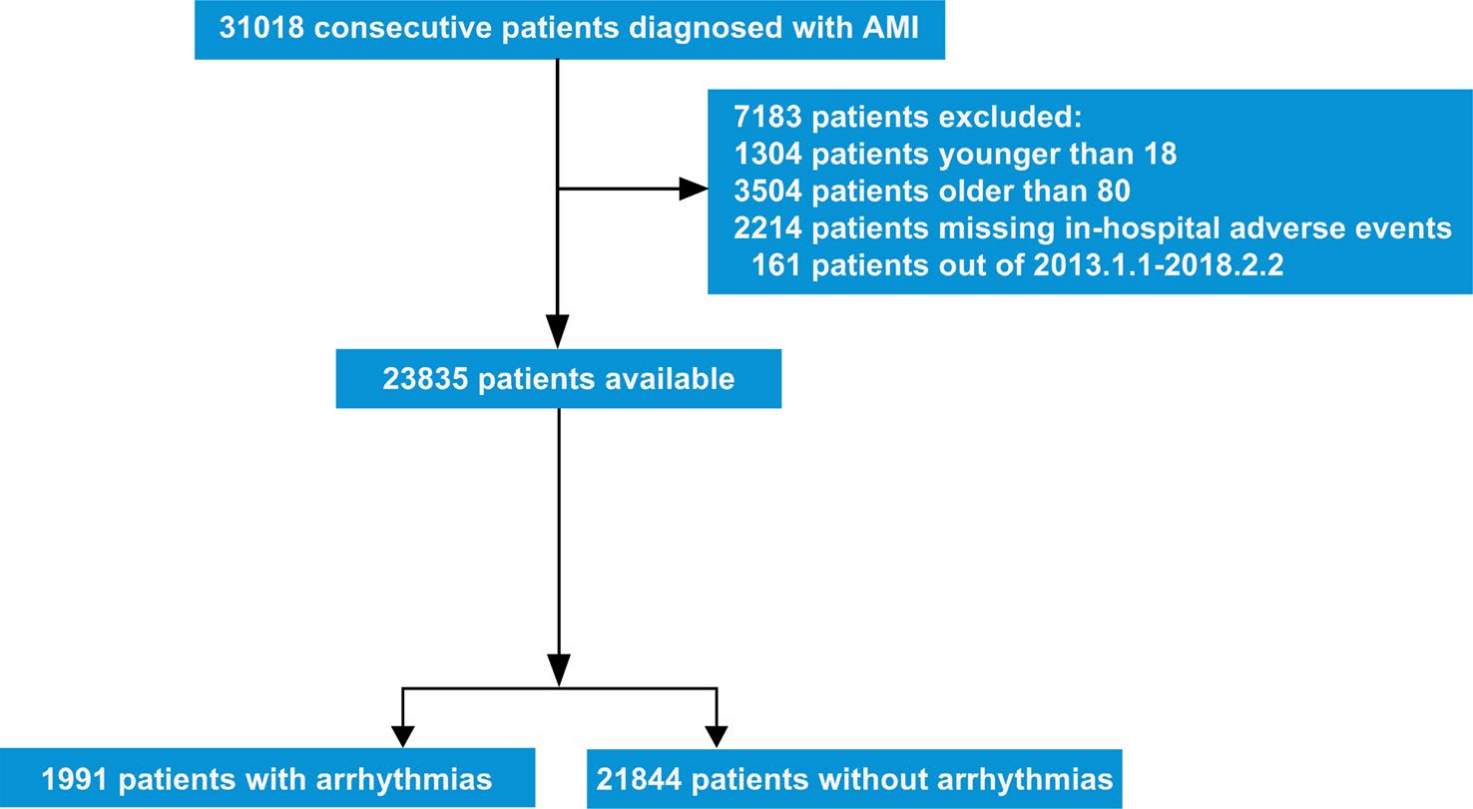
The flowchart for enrollment of patients. AMI = acute myocardial infarction

**Fig. 2:**
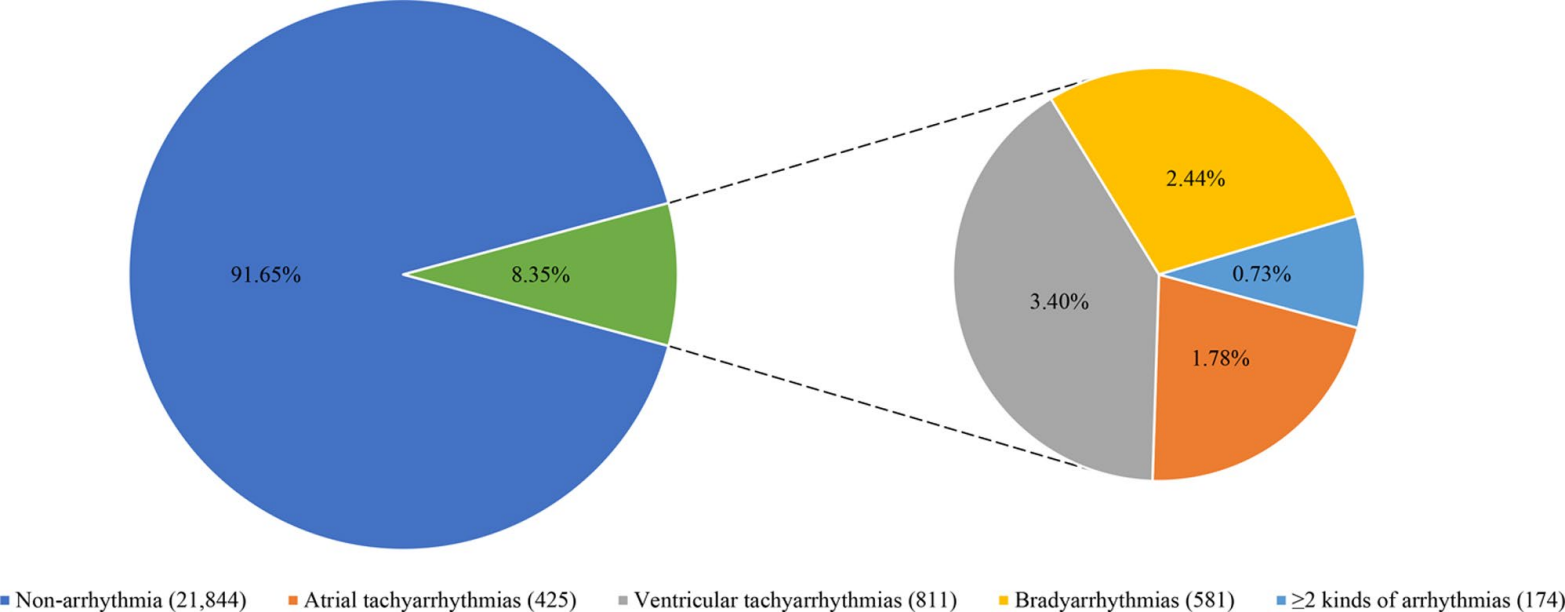
The distribution of all types of cardiac arrhythmias in AMI patients

A total of 23,835 patients, including STEMI and NSTEMI, were included in our analysis. Figure 1 is the flowchart for patient enrollment. In total, cardiac arrhythmias were presented in 8.35% (1,991/23,835) of AMI patients during hospitalization. Figure 2 shows the distribution of all kinds of cardiac arrhythmias. Ventricular tachyarrhythmias were the most common cardiac arrhythmias accounting for 3.40%. The incidence of bradyarrhythmias and atrial tachyarrhythmias were 2.44% and 1.78%, respectively. Only 0.73% of patients presented with ≥ 2 kinds of above arrhythmias.

### Clinical characteristics of AMI patients with cardiac arrhythmias

The baseline clinical characteristics of the study population are summarized in Table 1. Compared with those without arrhythmias, patients with arrhythmias were older (62.92 ± 11.26 vs. 60.22 ± 11.28, *P* < 0.001), had lower body mass index (BMI) (24.02 ± 3.04 vs. 24.35 ± 3.14, *P* < 0.001), had higher prevalence of chronic kidney diseases (1.6% vs. 0.9%, *P* = 0.007), previous heart failure (3.7% vs. 1.5%, *P* < 0.001) and previous stroke (12.5% ​​vs. 7.8%, *P* < 0.001). The proportion of STEMI (83.3% vs. 75.5%, *P* < 0.001) and worsening cardiac function (Killip class III-IV, 15.0% vs. 3.8%, *P* < 0.001; LVEF, 51.57 ± 10.75 vs. 54.30 ± 9.88, *P* < 0.001) were also higher in patients with arrhythmias. The proportion of patients undergoing PCI and CABG was similar between patients with and without arrhythmias. Patients with arrhythmia were more commonly receiving fibrinolysis therapy than those without arrhythmias (12.8% vs. 8.0%, *P* < 0.001).

**Table 1:** Baseline characteristics of cohorts with or without arrhythmias

	Total (*N* = 23,835)			Arrhythmias (*N* = 1,991)				
	Non-arrhythmia (*N* = 21,844)	Arrhythmias (*N* = 1,991)	P value	Atrial tachyarrhythmias (*N* = 425)	Ventricular tachyarrhythmias (*N* = 811)	Bradyarrhythmias (*N* = 581)	≥ 2 kinds of arrhythmias (*N* = 174)	*P* value
Age (yrs)	60.22 ± 11.28	62.92 ± 11.26	< 0.001	66.99 ± 9.45	59.67 ± 11.65	64.20 ± 10.59	63.86 ± 11.42	< 0.001
Male (n, %)	16,868 (77.2%)	1,516 (76.1%)	0.28	304 (71.5%)	652 (80.4%)	427 (73.5%)	133 (76.4%)	0.002
BMI	24.35 ± 3.14	24.02 ± 3.04	< 0.001	23.80 ± 2.80	24.15 ± 3.13	24.06 ± 3.12	23.82 ± 2.90	0.21
Current smoker (n, %)	10,448 (48.1%)	896 (45.1%)	0.01	151 (35.5%)	414 (51.0%)	249 (43.1%)	82 (47.1%)	< 0.001
Family history (n, %)	853 (3.9%)	73 (3.7%)	0.58	12 (2.8%)	41 (5.1%)	18 (3.1%)	2 (1.1%)	0.02
Comorbidities								
Hypertension (n, %)	10,459 (48.0%)	995 (50.1%)	0.08	225 (52.9%)	392 (48.4%)	301 (52.0%)	77 (44.3%)	0.14
Hyperlipidemia (n, %)	1,934 (8.9%)	125 (6.3%)	< 0.001	18 (4.2%)	57 (7.0%)	44 (7.6%)	6 (3.4%)	0.04
Diabetes mellitus (n, %)	4,041 (18.6%)	373 (18.8%)	0.82	87 (20.5%)	125 (15.5%)	127 (22.0%)	34 (19.5%)	0.01
CKD (n, %)	202 (0.9%)	32 (1.6%)	0.007	5 (1.2%)	15 (1.9%)	5 (0.9%)	7 (4.0%)	0.05
Medical History								
Previous angina pectoris (n, %)	4,925 (26.1%)	480 (26.1%)	0.97	108 (27.7%)	203 (27.0%)	127 (23.6%)	42 (26.3%)	0.45
Previous PCI/CABG (n, %)	1,080 (5.7%)	95 (5.2%)	0.33	18 (4.6%)	47 (6.3%)	21 (3.9%)	9 (5.6%)	0.27
Previous MI (n, %)	1,439 (7.6%)	170 (9.0%)	0.02	33 (8.5%)	91 (12.1%)	31 (5.8%)	15 (9.3%)	< 0.001
Previous HF (n, %)	331 (1.5%)	73 (3.7%)	< 0.001	24 (5.6%)	26 (3.2%)	14 (2.4%)	9 (5.2%)	0.04
Previous Stroke (n, %)	1,719 (7.9%)	248 (12.5%)	< 0.001	68 (16.0%)	79 (9.8%)	82 (14.2%)	19 (10.9%)	0.007
Previous PAD (n, %)	141 (0.6%)	8 (0.4%)	0.16	2 (0.5%)	2 (0.2%)	1 (0.2%)	3 (1.7%)	0.12
Clinical characteristics								
STEMI (n, %)	16,487 (75.5%)	1,658 (83.3%)	< 0.001	299 (70.4%)	697 (85.9%)	517 (89.0%)	145 (83.3%)	< 0.001
LVEF (%)	54.30 ± 9.88	51.57 ± 10.75	< 0.001	50.57 ± 11.15	50.16 ± 10.81	54.89 ± 9.45	49.72 ± 11.31	< 0.001
LVEDD (mm)	49.17 ± 12.07	49.82 ± 9.07	0.01	50.44 ± 9.87	49.87 ± 9.75	48.96 ± 7.60	51.05 ± 7.41	0.07
Killip classification III-IV (n, %)	832 (3.8%)	297 (15.0%)	< 0.001	62 (14.6%)	120 (14.9%)	76 (13.1%)	39 (22.4%)	0.04
Treatments								
PCI (n, %)	8,306 (38.0%)	800 (40.2%)	0.27	130 (30.6%)	374 (46.1%)	234 (40.3%)	62 (35.6%)	0.18
CABG (n, %)	25 (0.1%)	2 (0.1%)	0.92	0	1 (0.1%)	1 (0.2%)	0	0.73
Fibrinolysis (n, %)	1,752 (8.0%)	254 (12.8%)	< 0.001	34 (8.0%)	128 (15.8%)	69 (11.9%)	23 (13.2%)	0.28

Data are reported as mean ± SD.

Abbreviation: BMI=body mass index; CKD=chronic kidney disease; PCI=percutaneous coronary intervention; CABG=coronary artery bypass graft; MI=myocardial infarction; HF=heart failure; PAD=peripheral artery disease; STEMI= ST-segment elevation myocardial infarction; LVEF=left ventricular ejection fraction; LVEDD= Left ventricular end-diastolic dimension.

The differences in clinical characteristics were also observed among patients with different specific classes of arrhythmias. Patients with ventricular tachyarrhythmias were the youngest (59.67 ± 11.65) and predominantly male (80.4%) and had a higher prevalence of current smokers (51.0%), previous MI history (12.1%) and lower prevalence of previous stroke history than patients with other arrhythmias (all *P* < 0.01). Bradyarrhythmia patients had the highest proportion of hyperlipidemia (7.6%) and diabetes history (22.0%) and the highest proportion with STEMI (89.0%) (all *P* < 0.05). Patients with atrial tachyarrhythmias were the oldest (66.99 ± 9.45) and had the highest prevalence of previous stroke (16%) and heart failure history (5.6%) than those with other kinds of arrhythmias (all *P* < 0.05). Patients with ≥ 2 kinds of arrhythmias exhibited the lowest left ventricular ejection fraction (LVEF 49.72 ± 11.31) than those with other arrhythmias. The revascularization strategies, including PCI or CABG, were comparable among patients with different kinds of arrhythmias.

### The incidence of in-hospital outcomes in patients with cardiac arrhythmias

**Fig. 3:**
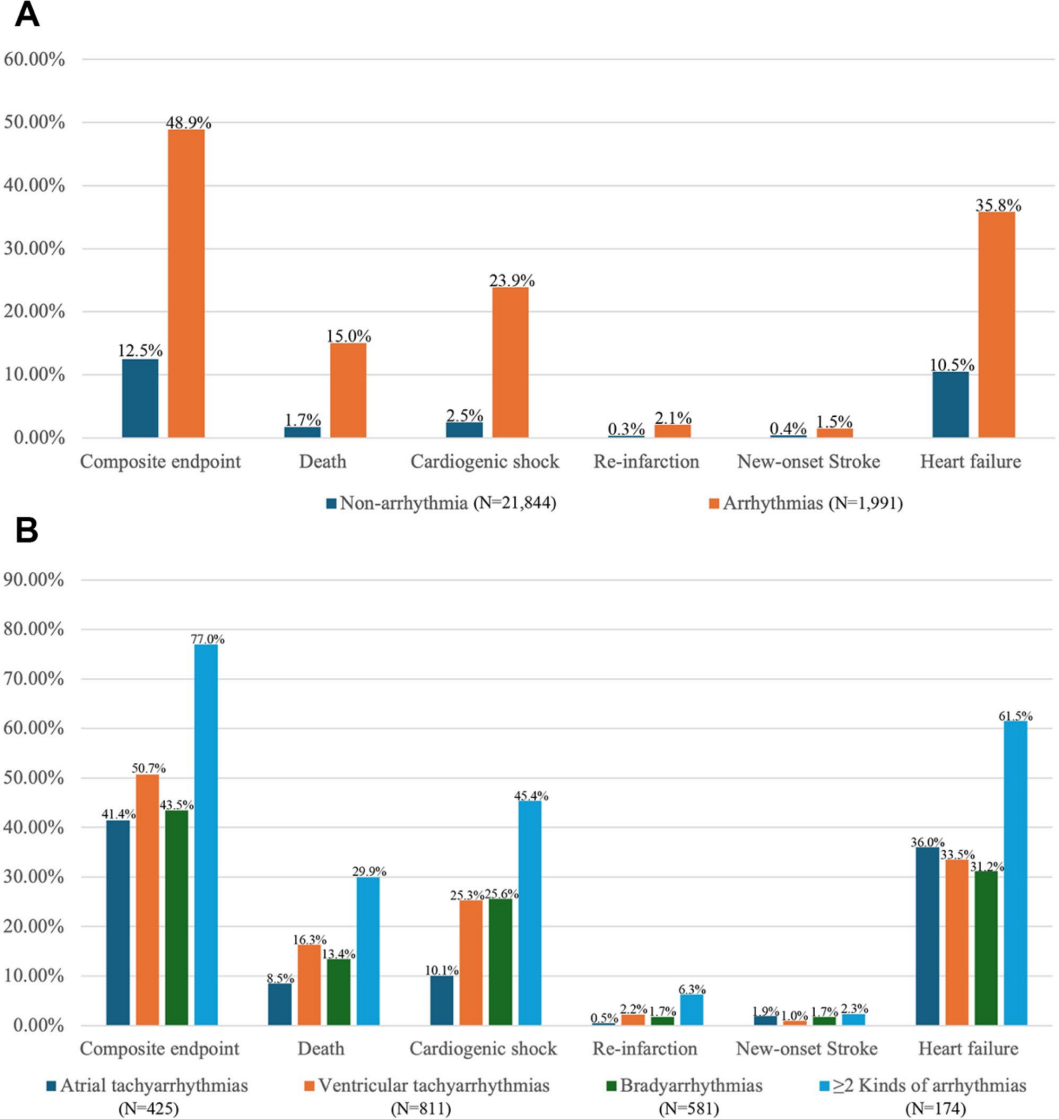
In-hospital clinical outcomes of cohorts with or without arrhythmias. Part A shows the distribution of adverse in-hospital clinical outcomes among patients with and without arrhythmias. Part B shows the distribution of adverse in-hospital clinical outcomes among patients with different arrhythmias

Figure 3 summarizes the in-hospital outcomes in this study population. The total in-hospital outcomes were 15.5% (3,697/23,835) and were higher in patients with cardiac arrhythmias than those without arrhythmias (48.9% vs. 12.5%, *P* < 0.001). In addition, patients with any arrhythmia presented higher death (15.0% vs. 1.7%, *P* < 0.001), cardiogenic shock (23.9% vs. 2.5%, *P* < 0.001), re-infarction (2.1% vs. 0.3%, *P* < 0.001), stroke (1.5% vs. 0.4%, *P* < 0.001) and heart failure (35.8% vs. 10.5%, *P* < 0.001) than those without arrhythmias. When in-hospital outcomes were compared among different arrhythmias, patients with ≥ 2 kinds of arrhythmias had the highest incidence of in-hospital composite outcomes, followed by patients with ventricular arrhythmias, then the bradyarrhythmias and atrial arrhythmias (77.0%, 50.7%, 43.5%, 41.4%, respectively). Similar trends were observed in the incidence of death and re-infarction among patients with different cardiac arrhythmias. The incidences of cardiogenic shock in patients with ventricular arrhythmias were similar to that in patients with bradyarrhythmias, lower than that in patients with ≥ 2 kinds of arrhythmias, and higher than that in patients with atrial arrhythmias. The incidence of new-onset stroke and heart failure was highest in patients with ≥ 2 kinds of arrhythmias, followed by patients with atrial arrhythmias.

### The impact of cardiac arrhythmias on in-hospital outcomes in patients with AMI

**Fig. 4:**
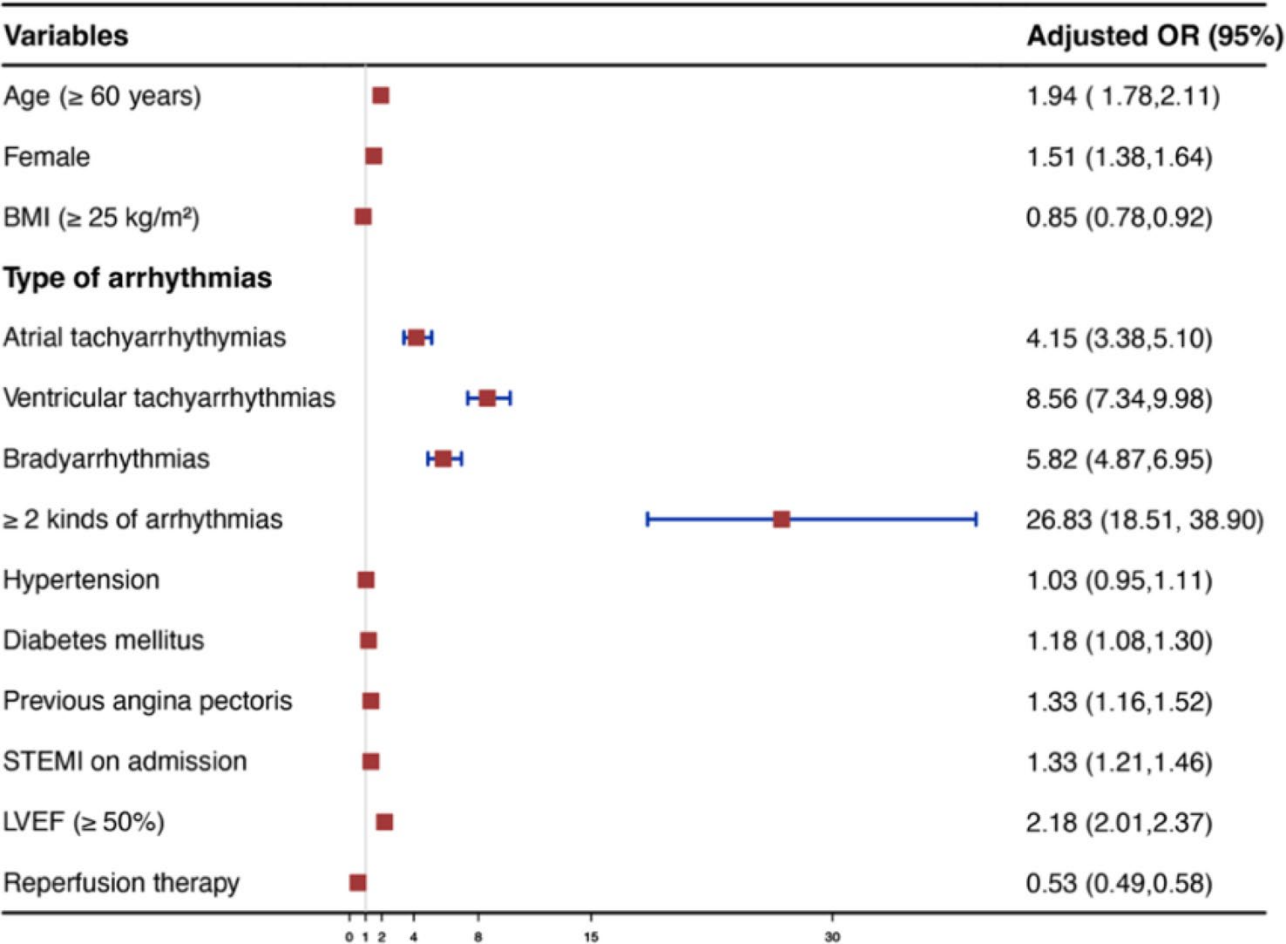
Multivariable regression analysis for risk factors of in-hospital composite endpoint events. In-hospital composite endpoint events included death, cardiogenic shock, cardiac arrest, re-infarction, new-onset stroke, or heart failure. Abbreviation: BMI, Body Mass Index; STEMI, ST-segment elevation myocardial infarction; LVEF, left ventricular ejection fraction

**Fig. 5:**
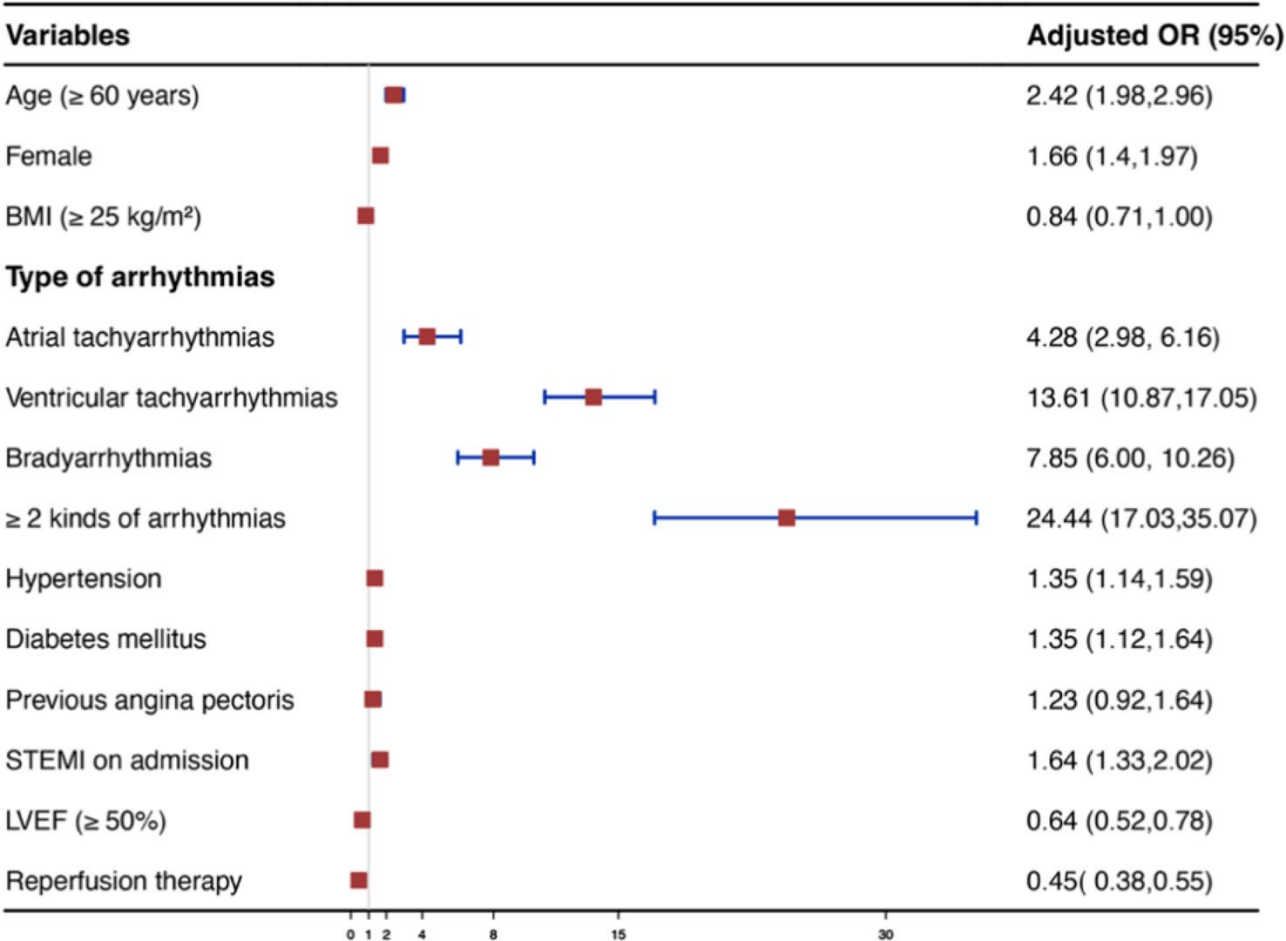
Multivariable regression analysis for risk factors of in-hospital mortality. Abbreviation: BMI = Body Mass Index; STEMI = ST-segment elevation myocardial infarction; LVEF = left ventricular ejection fraction

**Fig. 6:**
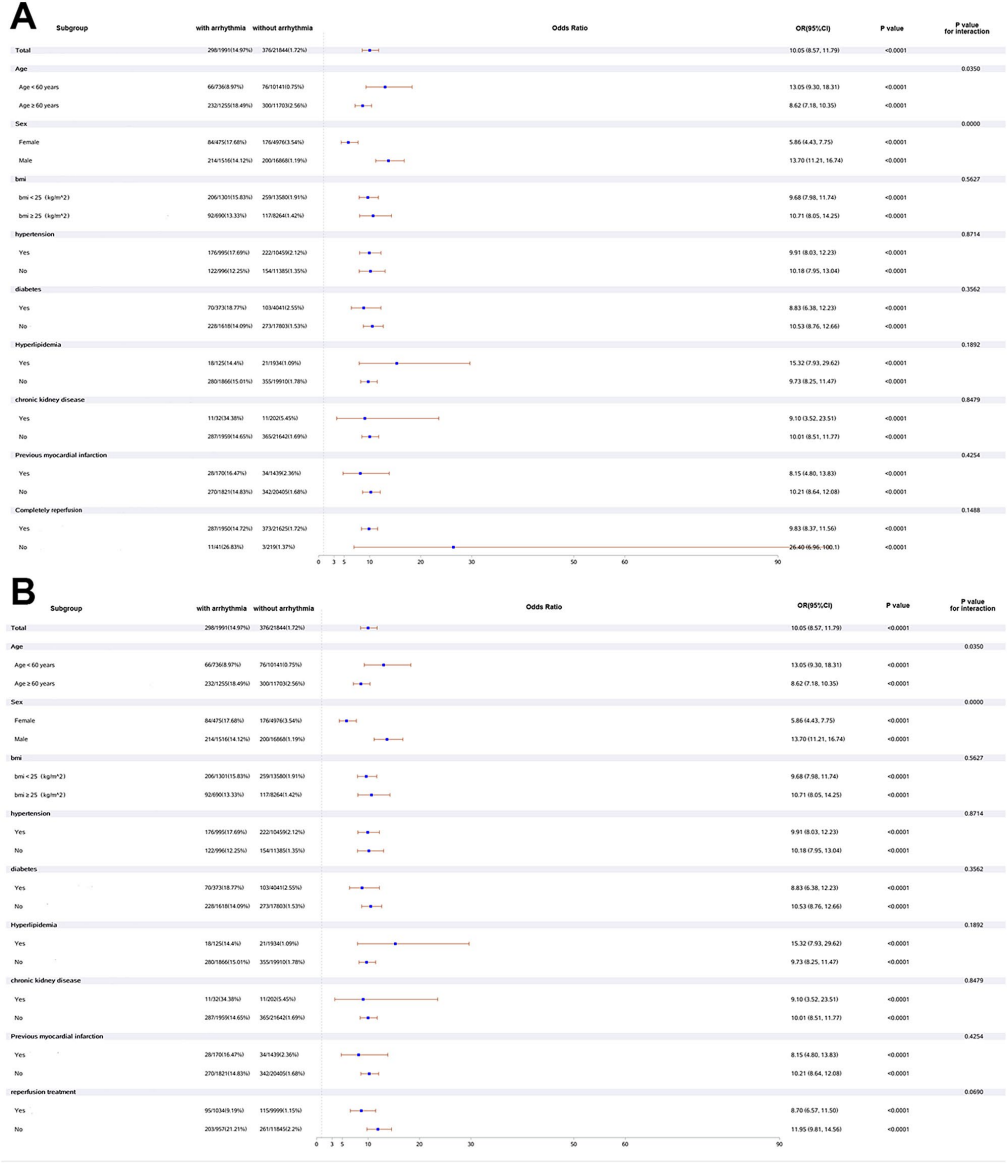
Subgroup analysis for the impact of cardiac arrhythmia on in-hospital mortality. Complete reperfusion is defined as post-PCI TIMI flow = 3, and incomplete reperfusion is defined as post-PCI TIMI flow = 0–2

Figure 4 shows a multivariate logistic regression analysis of risk factors for the in-hospital outcomes. The presence of any arrhythmia was independently associated with an increased risk of in-hospital outcomes in patients with AMI. Patients with ≥ 2 kinds of arrhythmias had the highest increased risk for a composite of in-hospital outcomes (OR 26.83, 95%CI 18.51–38.90). For a single specific class of arrhythmia, the risk of composite in-hospital outcomes was progressively increased in patients with atrial tachyarrhythmias (OR 4.15, 95%CI 3.38–5.10), bradyarrhythmias (OR 5.82, 95%CI 4.87–6.95), and ventricular tachyarrhythmias (OR 8.56, 95%CI 7.34–9.98). Figure 5 shows the association of all kinds of cardiac arrhythmias with in-hospital mortality in patients with AMI. A similar trend of progressive increase in the impact of cardiac arrhythmias was observed on the risk of in-hospital mortality: atrial tachyarrhythmias (OR 4.28, 95%CI 2.98–6.16); bradyarrhythmias (OR 7.85, 95%CI 6.00-10.26); ventricular tachyarrhythmias (OR 13.61, 95%CI 10.87–17.05); and ≥ 2 kinds of arrhythmias (OR 24.44, 95%CI 17.03–35.07). The association between arrhythmia and in-hospital mortality was consistent among most subgroups except in different genders and different ages (Fig. 6). Most importantly, either complete reperfusion or reperfusion treatment did not alter the overall trend. Among patients with complete reperfusion, those with arrhythmia had higher in-hospital mortality compared to those without arrhythmia (14.72% vs. 1.72%; OR 9.83, 95% CI 8.37–11.56). Similarly, in the group of patients with incomplete reperfusion, in-hospital mortality was higher in those with arrhythmia compared to those without arrhythmia (26.83% vs. 1.37%; OR 26.40, 95% CI 6.96–100.1, P for interaction = 0.1488). Among patients with reperfusion treatment, patients with arrhythmia were associated with higher in-hospital mortality than patients without arrhythmia (9.19% vs. 1.15%; OR 8.70, 95%Cl 6.57–11.50). Similarly, in the group of patients without reperfusion treatment, in-hospital mortality ranged from 21.21% in those with arrhythmia to 2.2% in those without arrhythmia (OR 11.95, 95%Cl 9.81–14.56, P for interaction = 0.0690).

## Discussion

The present study demonstrated the prevalence of three specific classes of cardiac arrhythmias and the combination of ≥ 2 kinds of cardiac arrhythmias in patients with AMI. The incidence of ventricular tachyarrhythmias, bradyarrhythmias, and atrial tachyarrhythmias might decline in turn in patients with AMI. Few patients (0.73%) presented with ≥ 2 kinds of arrhythmias. The presence of any cardiac arrhythmias in patients with AMI could impact on high risk of composite in-hospital outcomes and in-hospital mortality. The risk of composite in-hospital outcomes or in-hospital mortality was sequentially increased in AMI patients with atrial tachyarrhythmias alone, bradyarrhythmias alone, ventricular tachyarrhythmias alone, and with ≥ 2 kinds of arrhythmias when compared with those without any kinds of arrhythmias.

Previous studies have reported varying incidences of cardiac arrhythmia in AMI patients, approximately atrial tachyarrhythmias at 6-21% [7], ventricular tachyarrhythmias at 5-10% [18], and bradyarrhythmias at 3–7% [12]. The prevalence of cardiac arrhythmias during the natural course of AMI has declined over time due to advancements in early reperfusion techniques and medical therapy [19]. Thomsen et al. first conducted the long-term recording of cardiac arrhythmias in patients after myocardial infarction [20]. They documented a 28% incidence of new-onset atrial fibrillation, a 22% incidence of severe bradycardia (10% high-degree atrioventricular block, 7% sinus bradycardia, 5% sinus arrest), and a 6% incidence of ventricular tachyarrhythmias (3% sustained ventricular tachycardia, 3% ventricular fibrillation). However, the population included in their study was limited to patients with reduced LVEF ≤ 40%. Vallabhajosyula et al. reported on the prevalence of arrhythmias in AMI patients complicated with cardiogenic shock. The prognostic impact of all types of arrhythmias was evaluated, and they found the development of cardiac arrhythmias during hospitalization in patients with AMI was associated with worse acute organ failure and greater resource utilization but not associated with higher mortality [21]. Compared with previous results, our study demonstrated relatively lower incidences of general cardiac arrhythmias (8.35%), as well as the specific class of atrial tachyarrhythmias (1.78%), ventricular tachyarrhythmias (3.40%), and bradyarrhythmias (2.44%), and ≥ 2 kinds of arrhythmias (0.73%) in Chinese AMI patients. Possible reasons for the relatively low prevalence of cardiac arrhythmias in our study population might be the following: (1) our study population was general adults with AMI instead of AMI patients with heart failure or elderly patients. Advanced age is considered the most important impacting factor for atrial fibrillation, sick sinus syndrome, and other cardiac arrhythmias. According to our study aim, we excluded patients over 80 years old to ensure less confounding of general cardiac arrhythmias in the elderly. (2) a relatively high proportion of AMI patients in our study received reperfusion therapy. Among the population included in our study, AMI patients receiving PCI, CABG, and fibrinolysis are 9,106 (38.2%), 27 (0.11%), and 2,006 (8.41%), respectively. The proportion of AMI patients receiving reperfusion therapy reported in the previous studies ranges between 15.8% [22] and 45.2% [23]. The incidence of arrhythmia after AMI has declined in the contemporary era, suggesting that timely reperfusion therapy may potentially reduce the occurrence of cardiac arrhythmias.

The clinical characteristics of AMI patients with arrhythmias in our study confirmed previous clinical predisposing factors for the occurrence of cardiac arrhythmias. Arrhythmias were more common in patients with STEMI or receiving fibrinolysis therapy. STEMI patients frequently experience sudden onset and greater myocardial damage and are more likely to develop ventricular arrhythmias [24]. The fibrinolysis treatment could increase the susceptibility to developing arrhythmias due to reperfusion injury to the myocardium [25]. Our study revealed that AMI patients with arrhythmias showed a higher prevalence of chronic kidney diseases, previous heart failure, and previous stroke. Whether these factors could predispose AMI patients to the occurrence of cardiac arrhythmias needs further research. In addition, our study found no significant difference in revascularization strategies, including PCI and CABG, between AMI patients with or without arrhythmias, which was consistent with findings from other studies on patients with NSTEMI [26] and STEMI [27].

Previous studies have reported the impact of several arrhythmias on AMI patient’s clinical outcomes. Atrial fibrillation [28], ventricular arrhythmias [29], or atrioventricular block [12] have already been proven to be associated with a higher risk of short-term and long-term mortality in patients with AMI. However, these studies only focused on one specific single class of arrhythmias. To our knowledge, our study was the first to comprehensively assess the impact of three kinds of arrhythmias alone and the combination of two or more kinds of arrhythmias on in-hospital clinical outcomes in patients with AMI. Our results indicate that the risk of composite in-hospital outcomes and in-hospital mortality might be different among AMI patients with different cardiac arrhythmias. Patients with ≥ 2 kinds of arrhythmias might be at the highest increased risk of in-hospital mortality and all kinds of adverse events. The risk of composite in-hospital outcomes or in-hospital mortality was sequentially increased in AMI patients with atrial tachyarrhythmias alone, bradyarrhythmias alone, and then ventricular tachyarrhythmias. In addition, our results also suggested AMI patients with different kinds of arrhythmias may be predisposed to different adverse events. We found that the incidences of cardiogenic shock in patients with ventricular arrhythmias or bradyarrhythmias were higher than in patients with atrial arrhythmias, while the incidence of new-onset stroke and heart failure was higher in patients with atrial arrhythmias than patients with bradyarrhythmias.

## Limitation

Several limitations of our study should be mentioned. Firstly, the types of arrhythmias were divided into three kinds of arrhythmias, not detailed specific classes due to the relatively low incidence of arrhythmias in our study population. However, our study is the first to prospectively investigate the prevalence of cardiac arrhythmias in Chinese patients with AMI. Our result provided new insight into the contemporary treatment strategies of AMI and their impact on in-hospital outcomes. Secondly, the presence of atrial arrhythmias or bradyarrhythmias might not be occurred after onset of AMI because these arrhythmias might be asymptomatic in some patients. However, it is difficult to make a refined judgment in any AMI population. And the low incidence of atrial arrhythmias or bradyarrhythmias in our study population may indicate less influence of the possible confounding. Thirdly, it is important to acknowledge the limitation stemming from the low standard reperfusion therapy rates, particularly in extrapolating the results to other developed countries. Further research and consideration of these factors would strengthen the broader applicability and implications of the findings. Moreover, the method of using only ECG to detect arrhythmia in our study might be considered weak and lead to an underestimated incidence of arrhythmia. However, our study population with AMI was routinely monitored using a wearable continuous ECG monitoring device during hospitalization. Whether symptomatic or asymptomatic sustained arrhythmia events occurred, 12 leads ECG were recorded to confirm the arrhythmia event. Therefore, the incidence of arrhythmia in our study was data from the real world, which may only underestimate non-sustained arrhythmia. A wearable continuous ECG monitoring device could detect sustained and non-sustained arrhythmia events in future studies.

## Conclusion

Ventricular tachyarrhythmias might be the most common cardiac arrhythmia, followed by bradyarrhythmias, atrial tachyarrhythmias, and ≥ 2 kinds of arrhythmias. The presence of any arrhythmias might impact poor hospitalization outcomes, particularly in cases with two or more kinds of arrhythmias.

## Data Availability

The data supporting this study’s findings are available from the corresponding author upon reasonable request. The corresponding author, Yuejin Yang (yangyj_fw@126.com), can be contacted to request permission to view the data.

## Abbreviations

BMI: Body Mass Index
CKD: Chronic Kidney Disease
PCI: Percutaneous Coronary Intervention
CABG: Coronary Artery Bypass Graft
MI: Myocardial Infarction
HF: Heart Failure
PAD: Peripheral Artery Disease
STEMI: ST-segment Elevation Myocardial Infarction
LVEF: Left Ventricular Ejection Fraction
LVEDD: Left Ventricular End-Diastolic Dimension
